# A Genotypic Analysis of Five *P. aeruginosa* Strains after Biofilm Infection by Phages Targeting Different Cell Surface Receptors

**DOI:** 10.3389/fmicb.2017.01229

**Published:** 2017-06-30

**Authors:** Diana P. Pires, Andreas Dötsch, Erin M. Anderson, Youai Hao, Cezar M. Khursigara, Joseph S. Lam, Sanna Sillankorva, Joana Azeredo

**Affiliations:** ^1^CEB-Centre of Biological Engineering, Universidade do MinhoBraga, Portugal; ^2^Institute of Functional Interfaces, Karlsruhe Institute of TechnologyEggenstein-Leopoldshafen, Germany; ^3^Department of Molecular and Cellular Biology, College of Biological Science, University of Guelph, GuelphON, Canada

**Keywords:** biofilms, bacteriophages, *P. aeruginosa*, bacterial resistance

## Abstract

Antibiotic resistance constitutes one of the most serious threats to the global public health and urgently requires new and effective solutions. Bacteriophages are bacterial viruses increasingly recognized as being good alternatives to traditional antibiotic therapies. In this study, the efficacy of phages, targeting different cell receptors, against *Pseudomonas aeruginosa* PAO1 biofilm and planktonic cell cultures was evaluated over the course of 48 h. Although significant reductions in the number of viable cells were achieved for both cases, the high level of adaptability of the bacteria in response to the selective pressure caused by phage treatment resulted in the emergence of phage-resistant variants. To further investigate the genetic makeup of phage-resistant variants isolated from biofilm infection experiments, some of these bacteria were selected for phenotypic and genotypic characterization. Whole genome sequencing was performed on five phage-resistant variants and all of them carried mutations affecting the *galU* gene as well as one of *pil* genes. The sequencing analysis further revealed that three of the *P. aeruginosa* PAO1 variants carry large deletions (>200 kbp) in their genomes. Complementation of the *galU* mutants with wild-type *galU* in *trans* restored LPS expression on the bacterial cell surface of these bacterial strains and rendered the complemented strains to be sensitive to phages. This provides unequivocal evidence that inactivation of *galU* function was associated with resistance to the phages that uses LPS as primary receptors. Overall, this work demonstrates that *P. aeruginosa* biofilms can survive phage attack and develop phage-resistant variants exhibiting defective LPS production and loss of type IV pili that are well adapted to the biofilm mode of growth.

## Introduction

*Pseudomonas aeruginosa* is a versatile opportunistic pathogen, which is considered one of the leading causes of hospital-acquired infections by gram-negative bacteria (Driscoll et al., 2007; Mesaros et al., 2007). Infections caused by *P. aeruginosa* are generally difficult to treat because this pathogen displays low susceptibility to a wide range of antibiotics as a result of intrinsic, adaptive and acquired resistance mechanisms (Wagner et al., 2008; Breidenstein et al., 2011). Furthermore, this bacterium also has an ability to adhere to surfaces and form biofilms, which makes it particularly difficult to eradicate due to the fact that the biofilm architecture forms a shell around a microbial community and confers to the microorganisms a protective environment (Drenkard, 2003; Mah et al., 2003; Høiby et al., 2010).

The antibacterial activity of phages against *P. aeruginosa* has been studied by various groups (Pires et al., 2015b). Results from *in vitro* (Fu et al., 2010; Pires et al., 2011; Torres-Barceló et al., 2014) and *in vivo* (Heo et al., 2009; Hawkins et al., 2010; Alemayehu et al., 2012; Fukuda et al., 2012) studies have shown that phage therapy constitutes an effective strategy to fight *P. aeruginosa* infections. Despite these encouraging results, phages and their bacterial hosts are constantly mingled in co-evolutionary processes and the bacteria have developed multiple strategies to survive despite the phage predation. These strategies include prevention of phage adsorption to the bacterial hosts, prevention of phage DNA entry by superinfection exclusion systems, cleavage of phage nucleic acids by restriction-modification systems or CRISPR-Cas systems, and death of the infected cell by abortive systems (Labrie et al., 2010). Consequently, the emergence of phage-resistant bacterial variants within a few hours after phage infection is almost unavoidable. Studies involving phage interaction with biofilms have reported a regrowth in the biofilm population after phage infection, which have been attributed to the development of phage-resistant variants (Fu et al., 2010; Pires et al., 2011). Nonetheless, few reports to date have focused on the study of phage-resistant populations within biofilms (Hosseinidoust et al., 2013b; Le et al., 2014). Oechslin et al. sequenced two phage-resistant *P. aeruginosa* strains and found mutations in genes encoding phage receptors, namely *pilT* and *galU* genes (Oechslin et al., 2016), but the mechanisms of phage resistance at a molecular level have not been well studied.

In this work, phages phiIBB-PAA2, vB_PaeM_CEB_DP1 and LUZ19 were tested, either individually or as a cocktail, to assess their interactions with *P. aeruginosa* PAO1 biofilms and planktonic cells. The emergence of phage-resistant variants was tracked during the course of the 48-h treatment. Based on phage susceptibility profiles, ten *P. aeruginosa* PAO1 variants isolated from the biofilm infection with the phage cocktail were selected for motility analysis, and the genomes of five of these variants were completely sequenced to understand their genetic profile.

## Materials and Methods

### Bacterial Strains, Bacteriophages and Culture Conditions

The reference strain *P. aeruginosa* PAO1 (DSM22644) is from the German Collection of Microorganisms and Cell Cultures. The bacterial strain was grown at 37°C in lysogeny broth (LB, also commonly called Luria Bertani medium), LB agar or LB soft agar overlays containing 0.6% (w/v) of agar. The three phages used in this work include phiIBB-PAA2 (Pires et al., 2014), vB_PaeM_CEB_DP1 (Pires et al., 2015a), and LUZ19 (Lavigne et al., 2013) (LUZ19 was kindly provided by Professor Rob Lavigne, KU Leuven, Belgium).

### Phage Production and Concentration

Phages were propagated using the double agar overlay technique (Azeredo et al., 2014). Briefly, 10 μL of isolated phage lysate was added to LB agar plates containing the host bacterial lawn and spread using a paper strip. The Petri dishes were then incubated at 37°C for 16–18 h. After incubation, 3–5 mL of saline magnesium buffer (SM buffer) (5.8 g/L NaCl, 2 g/L MgSO_4_ 7H_2_O, 50 mL/L 1 M Tris-HCl pH 7.5) were added to the lysate from a clear Petri dish and the plates were placed under agitation at 4°C for 18 h. Subsequently, the SM buffer with the eluted phages was collected, centrifuged (9,000 × *g*, 4°C, 10 min) and filtered (0.22 μm). Phage concentration was performed using established protocols described elsewhere (Azeredo et al., 2014). Briefly, 58.4 g/L of NaCl were added to the phage lysates and incubated at 4°C for 1 h under agitation (90 rpm). After incubation, this solution was centrifuged (9,000 × *g*, 4°C, 10 min) and the supernatant collected. Then, 100 g/L of PEG 8000 were mixed with the supernatant and incubated at 4°C for 16 h with agitation (90 rpm). The suspension was centrifuged again (9,000 × *g*, 4°C, 10 min), the supernatant discarded and the pellet resuspended in 5 mL of SM buffer per 50 mL of centrifuged sample. Chloroform was added in 1:4 (v/v) proportion and the suspension was centrifuged (3,500 × *g*, 4°C, 5 min). The aqueous phase (upper phase), which contained the phages was recovered, filtered (0.22 μm) and stored at 4°C until further use.

### Phage Titration

Plaque forming unit (PFU) counting was performed using double agar overlay technique (Kropinski et al., 2009). Serial dilutions of phage stock solutions in SM buffer were performed. Then, 100 μL of the diluted phage solution was mixed with 100 μL of the bacterial host and 3 mL of LB soft agar into a Petri dish already containing a layer of LB agar. The plates were incubated overnight at 37°C and the PFUs were counted.

### Biofilm Formation

Biofilms were formed on 96-well polystyrene microtiter plates (Orange Scientific) using established protocols (O’Toole and Kolter, 1998b; Friedman and Kolter, 2004) with minor modifications. *P. aeruginosa* PAO1 cultures were first grown for 16 h at 37°C and 120 rpm and then diluted 1:100 in LB. Each well was inoculated with 200 μL of this bacterial suspension and the microtiter plates were incubated in an orbital incubator for 24 h at 37°C and 120 rpm.

### Biofilm Infection with Phages

Biofilm infection experiments with phages phiIBB-PAA2, vB_PaeM_CEB_DP1 and LUZ19, individually or in three-phages cocktail, were performed using a multiplicity of infection (MOI) of 0.1 of each phage, according to a previously described protocol (Pires et al., 2013), with minor modifications. After biofilm formation for 24 h, the medium and planktonic bacteria were removed, and the wells were washed with fresh LB medium. Afterward, 200 μL of LB already containing the phage, the phage cocktail or the SM buffer (control) were added to each well. The plates were incubated at 37°C with agitation (120 rpm) and the colony forming units (CFU) were evaluated after 2, 4, 6, 8, 10, 12, 24, and 48 h of biofilm infection, as described below. For each time point three wells were surveyed.

### Quantification of Biofilm Viable Cells

The number of viable cells present in the biofilms were determined as previously described (Pires et al., 2013). Briefly, after the removal of growth medium, the wells were washed with saline [0.9% (w/v) NaCl]. Then, 200 μL of fresh saline was added to each well. After that, biofilms were scraped off and samples were collected and serially diluted in saline containing 5 mM of ferrous ammonium sulfate (FAS), to prevent further infection of bacteria by phage (McNerney et al., 2004; Swift et al., 2014; Alves et al., 2015). One drop (10 μL) was placed on a Petri dish and allowed to run down the plate, to obtain single colonies. CFU counts were carried out after overnight incubation at 37°C.

### Phage Infection of Planktonic Cultures

A *P. aeruginosa* PAO1 culture grown for 16 h was diluted 1:100 in a final volume of 25 mL and incubated for 24 h at 37°C and 120 rpm. After that time, cells were harvested by centrifugation (6,000 × *g*, 4°C, 5 min) and resuspended in fresh LB medium with phages or phage cocktail, at an MOI of 0.1. Control experiments were done using SM buffer instead of the phage. The suspensions were incubated at 37°C with agitation (120 rpm) and CFU counts were determined after 2, 4, 6, 8, 10, 12, 24, and 48 h, as for the biofilm infection experiments.

### Phage Susceptibility Assays

Nine single colonies from each time point were recovered in each independent experiment of biofilm and planktonic cell infection, and their susceptibility to the three phages was tested as described by Moons et al. (**Supplementary Figure S1**; Moons et al., 2013). The goal of this assay was to screen for bacterial resistance to phage infection, and select phage-resistant variants of *P. aeruginosa*, for posterior analysis. Phage susceptibility assays were also performed on the complemented strains that have restored *galU* function.

### Motility Assays

Swimming, swarming and twitching motilities were analyzed for ten *P. aeruginosa* PAO1 variants (isolated from 48-h biofilm infection with phage cocktail) using different media and protocols described elsewhere (Ha et al., 2014a,b; Turnbull and Whitchurch, 2014).

### Isolation of Bacterial Genomic DNA

Five *P. aeruginosa* PAO1 variants that displayed resistance to the three phages used in this study were chosen for genomic DNA isolation and subsequent sequencing. The *P. aeruginosa* cultures were grown overnight at 37°C and 1.5 mL of these bacterial suspensions were centrifuged (9,000 × *g*, 4°C, 5 min). The pellets were resuspended in 500 μL of extraction buffer (80 mM Tris pH 8.5, 200 mM NaCl, 0.5% SDS, 5 mM EDTA, 1 mg/mL Proteinase K) and incubated overnight at 65°C. After incubation, 500 μL of phenol:chloroform:isoamyl alcohol (25:24:1, v/v) were added and the tubes were placed under gently agitation for 10 min at room temperature. The solution was then centrifuged (13,000 × *g*, 4°C, 10 min) and the supernatant was transferred into new tubes. One volume of isopropyl alcohol 100% (v/v) was mixed with the recovered supernatant and the solution was centrifuged again (13,000 × *g*, 4°C, 15 min). The supernatant was discarded and the pellet was washed with ethanol 75% (v/v). After drying, the pellets were resuspended in sterile water and stored at -20°C.

### Library Preparation and Illumina Sequencing

DNA sequencing libraries were produced from 1 μg of genomic DNA, following the recommendations of the TruSeq DNA protocol (Illumina). Genomic DNA was sheared by sonication using a Covaris S2 instrument. Sizes and concentrations of DNA sequencing libraries were determined on a Bioanalyzer 2100 (DNA1000 chips, Agilent). Paired-end sequencing (2 × 50 bp) was performed on one lane on a Hiseq1500 (Illumina) platform using TruSeq PE Cluster KIT v3 – cBot – HS and TruSeq SBS KIT v3 – HS. Cluster detection and base calling were performed using RTAv1.13 and quality of reads assessed with CASAVA v1.8.1 (Illumina). The sequencing resulted in at least 25 million pairs of 50-nt long reads for each sample, with a mean Phred quality score > 35. The raw genomic sequence data of the individual samples is available from the European Nucleotide Archive (ENA^1^) under the accession number PRJEB15059.

### Genomic Analysis of Phage-Resistant Variants

The read pairs resulting from sequencing were mapped to the reference genome of *P. aeruginosa* PAO1 (RefSeq Accession No. NC_002516) using the Bowtie 2 aligner (Langmead and Salzberg, 2012), resulting in an overall alignment rate of at least 95% for each of the samples. Small sequence variations (SNPs and short insertions/deletions) were detected using SAMtools (Li, 2011) with a minimum read depth of five reads covering a putative variation site. Candidate sequence variations were inspected manually using Integrative Genomics Viewer (Thorvaldsdóttir et al., 2013) and compared with previously reported intra-strain variations in the *P. aeruginosa* PAO1 genome sequence (Dötsch et al., 2009; Klockgether et al., 2011) to identify and validate variations that are specific to the phage-resistant strains. Large deletions were detected by *de novo* genome assembly of the reads using IDBA-UD (Peng et al., 2012) in *hybrid* mode with the reference sequence of PAO1 as a template, and subsequently aligning the resulting contigs with the reference genome using Mauve (Darling et al., 2004).

### Preparation of LPS

The samples of bacterial LPS were prepared according to the protocol described by Hitchcock and Brown (H&B) (Hitchcock and Brown, 1983).

### SDS–PAGE and Western Immunoblotting of Bacterial LPS

Acrylamide running gels at 12% were prepared according to a modified Laemmli procedure with resolving gels devoid of SDS (Laemmli, 1970). Three microliter samples of H&B LPS, except for the O6 B-band blot in which the sample used was only 1 μL, were loaded on the acrylamide gel and run at 120 V for 100 min. Blotting was performed for 60 min at 200 mA. Skimmed milk (5%) in PBS was used to block the nitrocellulose (NC) blots (Rocchetta and Lam, 1997). Primary monoclonal antibodies (mAb) specific against *P. aeruginosa* LPS were from cell lines of mouse hybridoma, and the supernatants of the culture of these were used undiluted as described previously (Emara et al., 1995) to incubate with the NC blots overnight at room temperature. The mAb used included MF15-4 (serotype O5 B-band specific), MF83-1 (serotype O6 OSA specific), N1F10 (CPA specific), 5c-101 (outer-core specific), and 5c-7-4 (inner-core specific). Secondary antibodies used were goat anti-mouse F(ab)_2_-alkaline phosphatase conjugated and incubated at room temperature for 60 min. The blots were developed with the BCIP/NBT as per the manufacturer’s protocols (Sigma). The LPS banding patterns were visualized using the ultrafast silver nitrate-staining method that was described previously (Fomsgaard et al., 1990).

### TEM Observation

A single colony from a Tryptic Soy Agar plate was used to inoculate 5 mL of LB, and grown statically at 37°C for ∼68 h. Using a sterile inoculum loop a small amount (∼5 μL) of the pellicle that formed at the air-liquid interface was removed and resuspended in 50 μL of 0.1 M HEPES buffer pH 7.4. To disperse cellular clumps, the cell suspension was agitated/mixed by re-pipetting repetitively. Five microliter of the clarified suspensions were removed and applied to 200-mesh carbon-coated copper grids. The liquid was wicked away using filter paper (#1 Whatman) and stained with 5 μL of 2% (w/v) uranyl acetate (UA) for 30 s. The UA was wicked away and the grid was imaged using a single-tilt holder on an FEI Tecnai G2 F20 transmission electron microscope operating at an accelerating voltage of 200 kV and equipped with a bottom-mount Gatan 4k charge-coupled-device (CCD) camera.

### Construction of Complementation Strains

The *galU* gene was amplified by PCR from *P. aeruginosa* PAO1 genomic DNA using the primers 5′ CATGTCGAAGAATTCATGATCAAGAAATGTCTTTTC and 5′ CTTCATCGGGAACGGAAGCTTTCAGTGAGCCTTGCC. The resulting PCR product was digested with the restriction enzymes EcoRI/HindIII and ligated into the vector pHERD26T. The resulting construct pHERD26T::*galU* was verified by sequencing.

Chemically competent *P. aeruginosa* PAO1 (wild-type and phage-resistant variants) were prepared using the rubidium chloride method (Green and Rogers, 2013) with mid-log (OD_600_ 0.5-0.6) grown cells. Competent cells were stored at -80°C prior to use. Transformation of pHERD26T::*galU* into *P. aeruginosa* PAO1 occurred via heat shock at 42°C for 90 s followed by incubation in SOC media at 37°C with shaking at 200 rpm, for 1 h. Transformed cells were isolated on tryptic soy agar containing tetracycline at 100 μg/mL.

### Statistical Analysis

The statistical analysis of biofilm and planktonic cells infection experiments was done using two-way ANOVA with Bonferroni’s multiple comparisons test using GraphPad Prism 6. All tests were performed with a confidence level of 99%.

## Results

### Phage Infection of Biofilms and Planktonic Cells

Phage infection of *P. aeruginosa* PAO1 biofilms and planktonic cultures was performed over the course of 48 h with phages phiIBB-PAA2, vB_PaeM_CEB_DP1 and LUZ19, either individually or in a cocktail composed by the three phages. Samples for determining the number of CFU were taken every 2 h during the first 12 h of phage infection. After that, two more samples were taken at 24 and 48 h post biofilm treatment. This was performed to help us better understand the dynamics of phage infection and bacterial resistance over the course of experiment. We hypothesized that at the later time points the population is mostly comprised of *P. aeruginosa* phage-resistant variants.

**FIGURE 1:**
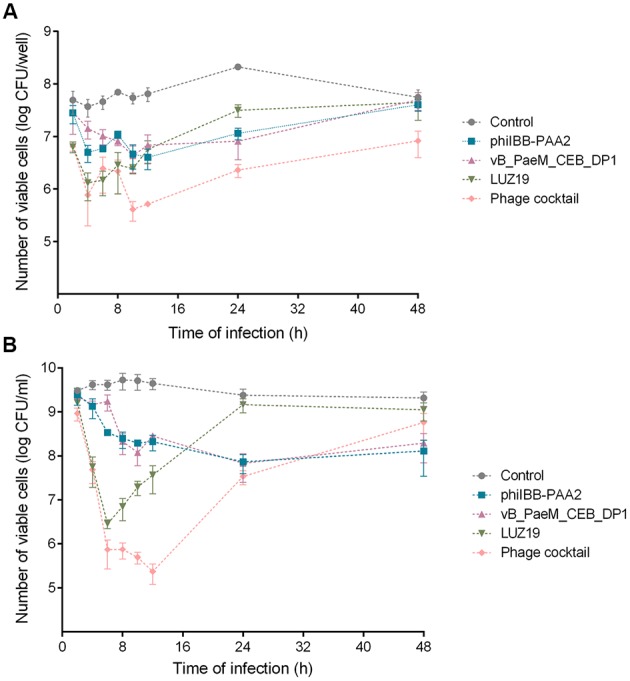
Infection of *Pseudomonas aeruginosa* PAO1 biofilms **(A)** and planktonic cultures **(B)** with phages phiIBB-PAA2, vB_PaeM_CEB_DP1 and LUZ19, individually or in cocktail. Both experiments were performed using a MOI of 0.1. Error bars represent standard deviations from three independent experiments performed in triplicate.

Overall, in the biofilm infection experiments (**Figure 1A**), phage LUZ19 was most effective among the phages tested in its ability to eliminate biofilm cells, followed by phages phiIBB-PAA2 and vB_PaeM_CEB_DP1. The latter two had similar levels of activity in depletion of *P. aeruginosa* biofilms. When the cocktail of the three phages was used, the results achieved during the first 8 h of biofilm infection were very similar with the ones obtained with phage LUZ19 alone. However, beyond 8 h, the phage cocktail was significantly more efficient (*p* < 0.01) than the other treatments with a particular phage in eliminating biofilm cells.

Although phage LUZ19 was able to significantly reduce the number of cells present in *P. aeruginosa* PAO1 biofilms, achieving a maximum reduction of ∼1.5 orders-of-magnitude after 6 h of treatment compared to the control (*p* < 0.01), a gradual increase in the number of biofilm cells was observed in subsequent time points (**Figure 1A**), which might indicate the fast proliferation of phage-resistant variants toward this phage. The use of the phage cocktail for biofilm treatment apparently resulted in two reduction phases: there is a reduction of ∼1.8 orders of magnitude within 4 h followed by a short period of regrowth, and then a maximum reduction of ∼2.1 orders-of-magnitude in the number of biofilm cells is achieved between 10 and 12 h of biofilm treatment versus the control (*p* < 0.01). This biphasic behavior might be a consequence of the early development of variants that get resistant to phage LUZ19, which has a shorter latent period and a higher burst size than the other two phages and consequently replicates faster in the first period of infection (Ceyssens et al., 2011; Pires et al., 2015a).

Although an increase in the number of biofilm cells was observed in the last time points, the number of viable cells present in biofilms 24 and 48 h after treatment with phage cocktail remained significantly low compared to the control (∼1.9 and ∼0.8 orders-of-magnitude reduction, respectively; *p* < 0.01) (**Figure 1A**).

The individual use of phages phiIBB-PAA2 and vB_PaeM_CEB_DP1 revealed to be the less effective treatments. The maximum reductions of biofilm cells achieved with those phages were ∼1.2 and ∼1.4 orders-of-magnitude after 12 and 24 h of biofilm infection, respectively (**Figure 1A**). Forty-eight hours post-infection of the biofilm, no statistic differences in the number of biofilm cells could be discerned when compared to the control.

In planktonic cells infection experiments, greater reductions in *P. aeruginosa* PAO1 cell counts were observed for all treatments when compared to that of the biofilm infection experiments (**Figure 1B**). Regarding planktonic cultures infection with phage LUZ19, a similar behavior as in biofilm infection experiments was observed: the maximum reduction of planktonic cells was achieved 6 h after phage treatment, showing a reduction of ∼3.2 order-of-magnitude vs. control (*p* < 0.01). Then, a gradual increase in the number of planktonic cells was observed in subsequent time points and no statistic differences were observed between the treatment with phage LUZ19 and the control after 24 and 48 h of infection (**Figure 1B**).

The application of a phage cocktail against planktonic cultures resulted in a fast reduction of cells in the first 6 h of infection followed by a slower reduction until a maximum reduction of ∼4.3 order-of-magnitude vs. control (*p* < 0.01) within 12 h. This reduction was followed by an increase in the number of planktonic cells and 48 h after treatment, no statistic differences comparatively with the control were detected (**Figure 1B**).

The individual treatment of planktonic cultures with phages phiIBB-PAA2 and vB_PaeM_CEB_DP1 resulted in a reduction of ca. 1.5 orders-of-magnitude vs. control (*p* < 0.01), which was obtained 24 h post-treatment. Although a slight increase in the number of planktonic cells was observed in the last time point for both treatments, the number of planktonic cells remained significantly lower comparatively with the control (*p* < 0.01) (**Figure 1B**).

### Phage Susceptibility of the *P. aeruginosa* PAO1 Variants

To determine the amount of time required post-treatment for the number of phage-resistant variants to begin to increase, ten colonies were isolated from each independent experiment, at each time point of biofilm and planktonic cells infection experiments and the isolates were characterized, particularly regarding their susceptibility to the phages.

**FIGURE 2:**
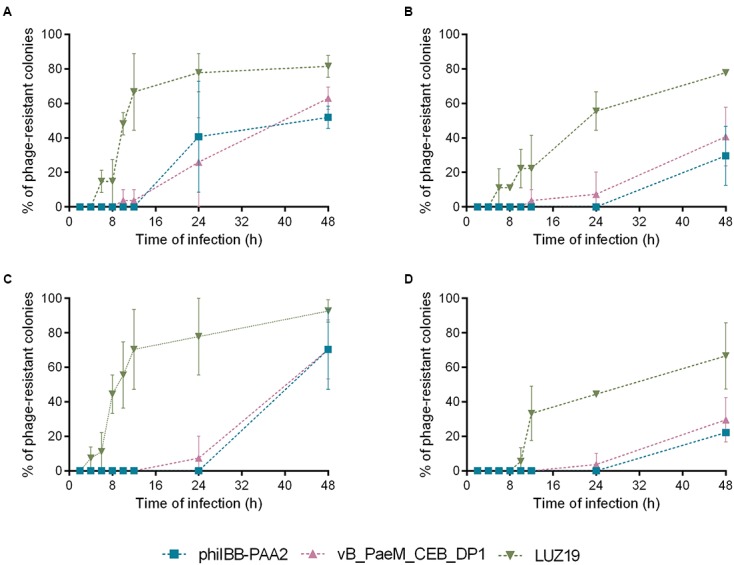
Percentage of *P. aeruginosa* PAO1 colonies isolated in each time point of infection experiments that were resistant to phages phiIBB-PAA2, vB_PaeM_CEB_DP1 and LUZ19. **(A)** Percentage of phage-resistant colonies isolated from biofilm infection experiments with each phage individually. **(B)** Percentage of phage-resistant colonies isolated from biofilm infection experiments with phage cocktail. **(C)** Percentage of phage-resistant colonies isolated from planktonic cultures infection experiments with each phage individually. **(D)** Percentage of phage-resistant colonies isolated from planktonic cultures infection experiments with phage cocktail. Error bars represent standard deviations from three independent experiments.

The percentages of phage-resistant variants isolated at each time point of the experiment were depicted in **Figure 2**. According to these results, the first phage-resistant variants were isolated from biofilm and planktonic cultures infected with phage LUZ19. *P. aeruginosa* PAO1 variants resistant to phage LUZ19 were isolated as early as 6 h after biofilm treatment with LUZ19 both individually (**Figure 2A**) and in cocktail (**Figure 2B**). In planktonic cultures, phage-resistant variants emerged within 4 h post-infection with LUZ19 (**Figure 2C**). However, when planktonic cultures were infected with phage cocktail, LUZ19-resistant variants were isolated 10 h post-treatment (**Figure 2D**). The emergence of resistant variants toward phages phiIBB-PAA2 and vB_PaeM_CEB_DP1 was significantly later compared to infection with phage LUZ19, i.e., between 12 and 24 h post-treatment of the biofilm (**Figures 2A,B**) and between 24 and 48 h post-infection of the planktonic cultures (**Figures 2C,D**). It noteworthy to point out that in both planktonic and biofilm infection experiments, the use of the cocktail of the three phages resulted in a lower percentage of phage-resistant variants than infection of the PAO1 cultures with individual phage.

Ten of the *P. aeruginosa* PAO1 variants, isolated after 48 h of biofilm treatment with the phage cocktail were selected for further characterization.

The susceptibility profiles of the ten *P. aeruginosa* variants to each phage are indicated in **Table 1**. *P. aeruginosa* PAO1 variants 1, 2, 8, and 9 showed resistance to phage LUZ19 but remained susceptible to phages phiIBB-PAA2 and vB-PaeM_CEB_DP1, whereas variants 3, 4, 5, 6, 7, and 10 were resistant to the three phages. These variants were streaked several times and their susceptibility profiles were maintained throughout the generations.

**Table 1:** Susceptibility of the *Pseudomonas aeruginosa* PAO1 wild-type and variant strains (1–10) to the phages phiIBB-PAA2, vB_PaeM_CEB_DP1 and LUZ19.

	phiIBB-PAA2	vB_PaeM_CEB_DP1	LUZ19
Wild-type	+	+	+
Variant 1	+	+	-
Variant 2	+	+	-
Variant 3	-	-	-
Variant 4	-	-	-
Variant 5	-	-	-
Variant 6	-	-	-
Variant 7	-	-	-
Variant 8	+	+	-
Variant 9	+	+	-
Variant 10	-	-	-

*+, Phage is able to induce plaque formation; -, Phage is not able to induce plaque formation.*

It was further observed that the colonies of variants 6, 7, and 10, all resistant to the three phages, had a brown color (data not shown), which is indicative of the production of a brown pigment, called pyomelanin, in the surrounding agar (Hosseinidoust et al., 2013b).

### Bacterial Motility

Overall, all ten of the selected *P. aeruginosa* PAO1 variants exhibited reduced twitching (*p* < 0.01) compared to the wild-type strain, which might indicate mutations in type IV pili of these variants. The swimming motility of all variant strains was also significantly inferior (*p* < 0.01) than the wild-type strain (**Table 2**). Concerning swarming motility, although all variant strains were statistically different from the wild-type strain, it increased in some cases and decreased in others. Therefore it was not possible to infer about the impact of phage resistance in swarming motility.

**Table 2:** Motility assays of *P. aeruginosa* PAO1 wild-type (wt) and variants (1–10) [avg. (±SD)] (three independent experiments were performed).

	Swimming motility (mm)	Swarming motility (mm)	Twitching motility (mm)
Wild-type	24.44 (2.55)	13.67 (1.00)	23 (2.65)
Variant 1	18.78 (2.78)*	11.00 (4.16)*	5.89 (0.51)*
Variant 2	18.67 (2.03)*	21.11 (2.59)*	5.44 (0.38)*
Variant 3	16.11 (3.29)*	10.11 (0.39)*	5.44 (0.19)*
Variant 4	12.22 (1.90)*	8.00 (1.20)*	5.22 (0.69)*
Variant 5	11.78 (0.39)*	18.78 (1.26)*	5.22 (0.84)*
Variant 6	5.00 (0.33)*	5.78 (0.51)*	4.78 (0.51)*
Variant 7	12.22 (0.77)*	8.00 (0.33)*	4.78 (0.51)*
Variant 8	13.68 (0.33)*	17.89 (0.69)*	5.78 (0.69)*
Variant 9	15.22 (0.84)*	17.89 (0.84)*	4.67 (0.88)*
Variant 10	8.44 (1.35)*	7.11 (0.69)*	4.67 (0.33)*

*^∗^Statistically different (p < 0.01) from the control (wild-type). All tests were performed using a confidence level of 99%.*

### Sequencing of Bacterial Genomic DNA

**FIGURE 3:**
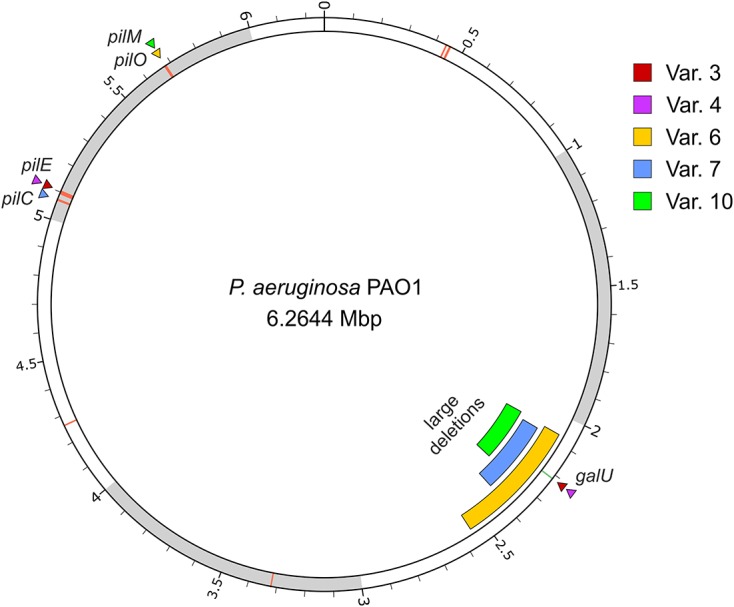
Location of the genomic variations of five phage-resistant strains derived from *P. aeruginosa*. The image displays the PAO1 wild-type chromosome with variant loci indicated by triangles (single nucleotide variants) or bars (large deletions). The location of the *pil* genes are additionally marked by red lines on the chromosome. The image was created using *circos* (Krzywinski et al., 2009).

The genomes of five *P. aeruginosa* PAO1 variant strains, which displayed resistance to the three phages used in this study and defects in motility were sequenced and compared to the wild-type genome in order to identify genetic variations specific to the resistance phenotype (**Figure 3** and Supplementary Table S1).

All variant strains were found to carry mutations in one of the *pil* genes involved in the synthesis of type IV pili: variants 3 and 4 carry a nonsense mutation in *pilE* gene, which encodes a pilin-like protein called “minor” pilin (Giltner et al., 2010; Kuchma et al., 2012); variant 6 carries a nonsense mutation in *pilO* gene, a gene required for pilin glycosylation (Castric, 1995); and variants 7 and 10 carry frameshift mutations in *pilC* and *pilM*, respectively, which encode a probable transmembrane protein PilC required to pilus biogenesis (Nunn et al., 1990; Li et al., 2013), and the cytoplasmic actin-like protein PilM that is likely anchored to the inner membrane by binding to the cytoplasmatic tail of PilN (Li et al., 2013; Tammam et al., 2013). Since there is an extended genomic diversity among *P. aeruginosa* PAO1 laboratory strains (Klockgether et al., 2010) and to confirm that these mutations were not a specification of the wild-type strain used in this study, the genes *pilE*, *pilO*, *pilC*, and *pilM* of wild-type strain were amplified and sequenced. The Sanger sequencing confirmed that all the mutations found in *pil* genes of variant strains were not already present in the wild-type strain, thus being acquired during the experiment (data not shown).

*Pseudomonas aeruginosa* variants 3 and 4 further revealed a point mutation in the *galU* gene, while in the other variants (6, 7, and 10), this gene was absent from the genome due to large deletions of 486, 317, and 214 kbp, respectively. Sanger sequencing of the wild-type *galU* confirmed that it is devoid of mutation when compared to that of the *P. aeruginosa* genome database (data not shown). Due to the size of these deletions, a large number of genes were lost from the genotypes of these strains (420, 266, and 179 genes, respectively, Supplementary Table S1).

Type IV pili are frequently described to play an important role in the initial stage of biofilm formation (O’Toole and Kolter, 1998a; Giltner et al., 2006, 2012; Barken et al., 2008), and thus the biofilm formation capability of *P. aeruginosa* PAO1 variant strains 3, 4, 6, 7, and 10 that were isolated from phage infection was analyzed. Nonetheless, the number of viable cells present in biofilms formed by those variants was similar to the numbers obtained with the parental strain and no statistical differences were observed (**Supplementary Figure S2**).

### LPS Analysis and TEM Visualization of *P. aeruginosa* PAO1 and Phage-Resistant Variants

**FIGURE 4:**
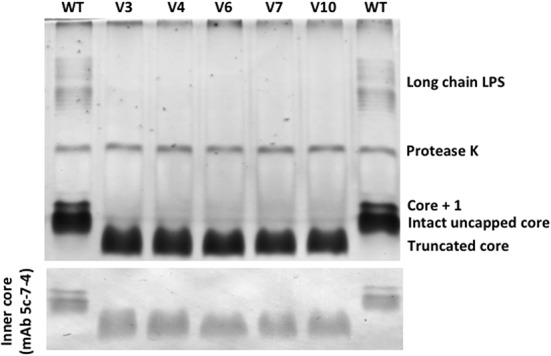
SDS–PAGE and Western immunoblots (probed with mAB 5c-7-4) of LPS from *P. aeruginosa* PAO1 wild-type (WT) and five phage-resistant variant strains (V3, V4, V6, V7, and V10).

Differences in the phenotype of LPS between the *P. aeruginosa* PAO1 wild-type strain and the phage-resistant variant strains were apparent (**Figure 4**). Comparing to the SDS–PAGE LPS banding profile of the wild-type *P. aeruginosa* PAO1 control, a fast-migrating core oligosaccharide band is observed in the five phage-resistant variants, indicating that all of these variant bacteria synthesize a truncated LPS core. Consequently, neither Common Polysaccharide Antigen (CPA) nor O-Specific Antigen (OSA) polysaccharide could be assembled onto the core and all variant strains also demonstrated a lack the long chain of LPS bands. This was verified by SDS–PAGE and Western immunoblotting using mAb N1F10 (specific for CPA) and MF15-4 (specific for OSA).

**FIGURE 5:**
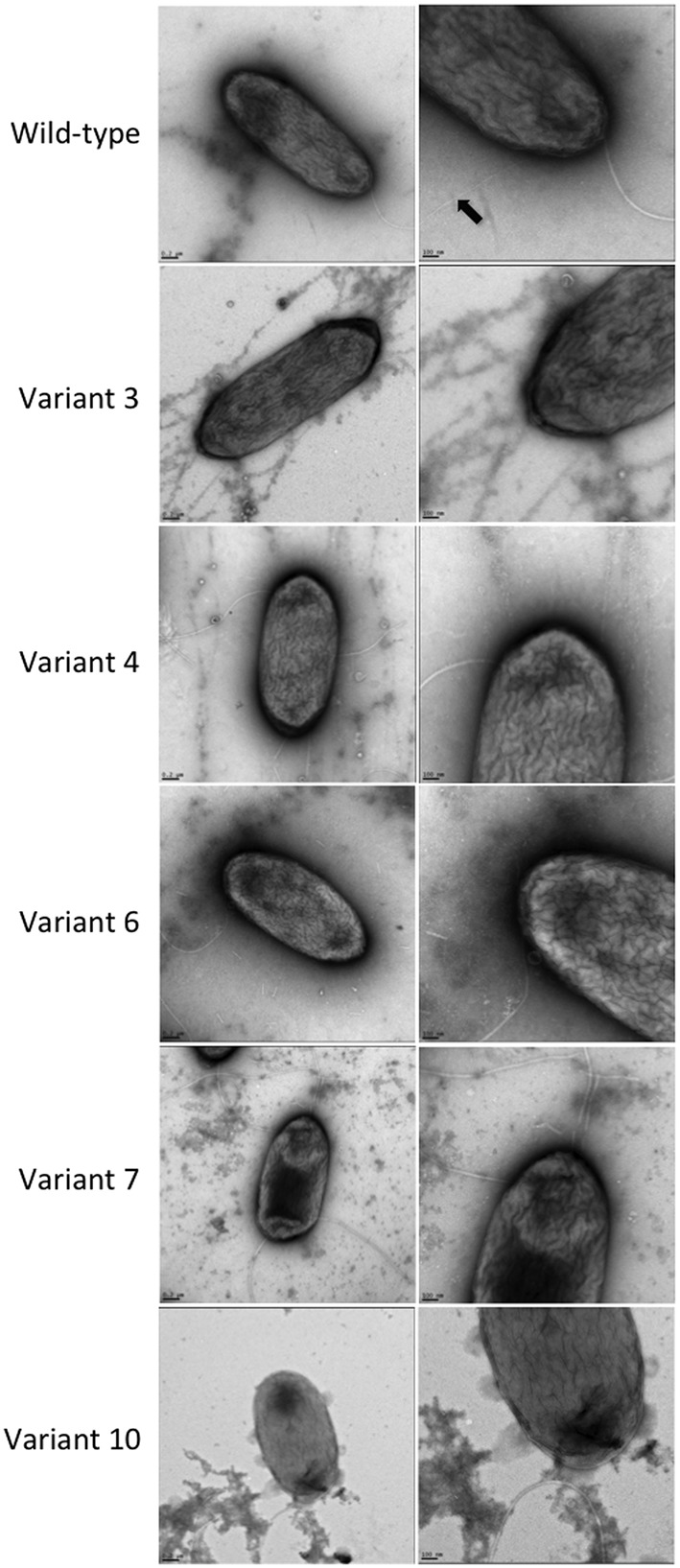
TEM observation of the *P. aeruginosa* PAO1 wild-type and five phage-resistant variant strains. A black arrow indicates the pili in the wild-type strain.

Electron micrographs obtained by TEM of the wild-type and variant strains, which were grown under static conditions, showed the presence of pili on the bacterial cells of the wild-type strain, but none could be discerned among the *P. aeruginosa* PAO1-derived phage-resistant variants (**Figure 5**).

### Complementation of *galU* Gene

To confirm that the loss of *galU* function was responsible for the acquisition of phage resistance by the variant strains, complementation of the *galU* gene in these variants was performed by transforming the *P. aeruginosa* PAO1 wild-type control and phage-resistant variants with pHERD26T::*galU* in *trans*. The susceptibility of the complemented bacterial strains to the three phages was evaluated. As anticipated, the susceptibility of the *P. aeruginosa* variant strains to phages phiBB-PAA2 and vB_PaeM_CEB_DP1 was restored but they remained resistant to phage LUZ19 (**Table 3**). Although these variants were complemented with *galU*, they still carry mutations in one of the *pil* genes and phage LUZ19 is reported to use type IV pili as primary receptors.

**Table 3:** Susceptibility of the *P. aeruginosa* PAO1 wild-type and phage-resistant variant strains (3, 4, 6, 7, and 10) complemented with *galU* gene to the phages phiIBB-PAA2, vB_PaeM_CEB_DP1 and LUZ19.

	phiIBB-PAA2	vB_PaeM_CEB_DP1	LUZ19
Wild-type + pHERD26T	+	+	+
Wild-type + pHERD26T::*galU*	+	+	+
Variant 3 + pHERD26T::*galU*	+	+	-
Variant 4 + pHERD26T::*galU*	+	+	-
Variant 6 + pHERD26T::*galU*	+	+	-
Variant 7 + pHERD26T::*galU*	+	+	-
Variant 10 + pHERD26T::*galU*	+	+	-

*+, Phage is able to induce plaque formation; -, Phage is not able to induce plaque formation.*

The LPS-banding profiles of the complemented strains were analyzed by SDS–PAGE and silver staining and they were practically identical to that of LPS prepared from the wild-type PAO1 strain (**Supplementary Figure S3**)

## Discussion

The efficacy of phages against biofilms has been widely studied and many research groups have reported on interesting results (Azeredo and Sutherland, 2008; Sillankorva et al., 2008, 2010; Donlan, 2009; Fu et al., 2010; Pires et al., 2011; Alemayehu et al., 2012; Alves et al., 2015). However, one of the major drawbacks of the application of phage therapy is associated with the relatively rapid emergence of phage-resistant variants. In many cases, after the initial reduction of bacterial cells caused by phage treatment, there is a gradual increase in the numbers of phage-resistant bacteria. For instance, the fast proliferation of phage-resistant variants after phage treatment of biofilms has been observed by Fu et al. (2010), who studied the effect of a pre-treatment of hydrogel-coated catheters with phages on the colonization by *P. aeruginosa* biofilms. In that study, hydrogel-coated catheters were first exposed to the *P. aeruginosa* phage M4 for 2 h followed by bacterial inoculation. Although a reduction of 2.8 orders of magnitude in the number of biofilm cells was observed after 24 h of biofilm formation in phage-treated catheters compared with untreated catheters, a regrowth of biofilms on phage-treated catheters was observed between 24 and 48 h of biofilm formation. This regrowth was attributed to the emergence of resistant variants to phage M4 in the pre-treated catheters (Fu et al., 2010). In a study by Hosseinidoust et al. (2013a), *P. aeruginosa* biofilms formed in microtiter plates pre-treated with phage E79 maintained levels of biomass significantly lower than the control for up to 24 h. After that time, an increase in biofilm biomass above the levels of the control was observed and the majority of the colonies isolated from biofilms showed resistance to phage E79. Nonetheless, this phenomenon has not been fully explored. In the present work, *P. aeruginosa* phage-resistant colonies were isolated as early as 6 h after biofilm treatment with phage LUZ19, which indicates the fast proliferation of phage-resistant cells after biofilm challenge.

One of the explanations to the development of phage resistance by bacteria is related to phage receptors. Bacteria can avoid phage predation by preventing phage adsorption (the initial step of phage infection) to their host receptors. This may happen due to changes in the structure of bacterial cell surface receptors or in their three-dimensional conformation (Labrie et al., 2010). It is known that type IV pili serve as primary receptors for LUZ19 phage (Lavigne et al., 2013), while the receptors of phage phiIBB-PAA2 and phage vB_PaeM_CEB_DP1 are believed to be in the LPS (Garbe et al., 2010; Pires et al., 2013). Thus, it is expected that phage-resistant variants suffered alterations in the composition of LPS and type IV pili, due to mutations or deletions of the genes encoding for such receptors.

In order to understand if the ten selected *P. aeruginosa* PAO1 variant strains, which were isolated from biofilm infection experiments with phage cocktail, were carrying mutations in genes encoding phage receptors, their motility profiles were analyzed. It is known that *P. aeruginosa* is able to perform different types of movement, including swimming, swarming, and twitching. Although it was not possible to understand the influence of phage resistance in swarming motility, all variant strains exhibited decreased swimming and twitching motilities as compared to the wild-type strain (**Table 2**). Similar results were observed in a study developed by Hosseinidoust et al. (2013b), where all *P. aeruginosa* variants obtained from different phage treatments showed decreased twitching motility. Swimming and swarming are flagellum-mediated motilities in which the first takes place as individual cells moving in liquid environments (Kearns, 2010) and the second is mediated by hyperflagellation of bacteria that thus move in a coordinated manner (Rashid and Kornberg, 2000; Harshey, 2003; Kearns, 2010). Although no genetic variations were observed in genes connected to the synthesis of flagella, the observed reduced swimming motility might be a secondary effect of the truncation of LPS core in phage-resistant variants. Some authors have reported that swarming is a rather complex type of motility since it is influenced by a large number of different genes (Overhage et al., 2007, 2008). For example, it was already shown that swarming of *P. aeruginosa* is dependent on both flagella and type IV pili (Kohler et al., 2000), and in addition influenced by the presence of rhamnolipids (Caiazza et al., 2005). Twitching motility is a flagellum-independent mode of surface translocation that requires type IV pili (Merz et al., 2000; Mattick, 2002). Consequently, the results obtained from motility analysis suggested possible mutations in genes encoding type IV pili, which could indeed be identified in each of five phage-resistant *P. aeruginosa* PAO1 variants that were analyzed by whole genome shotgun sequencing (**Figure 3**).

The sequencing results also revealed mutations or deletions of the *galU* gene, which is described to play an important role in the core synthesis of LPS (Dean, 2002). Therefore, *galU* mutants are devoid of O-antigen and have a truncated outer LPS core (Dean, 2002; Priebe et al., 2004), which is consistent with the results of the LPS analysis (**Figure 4**), confirming a functional inactivation of *galU* by deletion or point mutation in each of the analyzed variants. As confirmed by the complementation experiments, the inactivation or loss of *galU* gene was the cause of resistance acquisition by the variant strains to phages phiIBB-PAA2 and vB_PaeM_CEB_DP1, likely due to the function of LPS as a surface receptor for these phage particles. A similar observation was reported by Oechslin et al. (2016) who analyzed the genomic profile of two *P. aeruginosa* phage-resistant strains. When compared to the parental strain CHA, one of the mutant strains displayed a 15-bp deletion at the 3′ end of *pilT* gene, while the other mutant had a 362-kb deletion at a locus that include a gene such as *galU* (Oechslin et al., 2016).

Three of the *P. aeruginosa* variants lost the *galU* gene due to large deletions alongside many other genes. One of these genes is *hmgA*, which encodes the enzyme homogentisate-1,2-dioxygenase (Rodríguez-Rojas et al., 2009). The absence of this gene in variants 6, 7, and 10 corroborates the observation, already mentioned, that the colonies of these variants produced the brown pigment pyomelanin as a result from the accumulation, oxidation, and polymerization of homogentisic acid (Rodríguez-Rojas et al., 2009). The overproduction of pyomelanin by colonies subjected to phage challenge was already observed in other studies (Hosseinidoust et al., 2013b; Le et al., 2014), and has also been associated to the inactivation of the *hmgA* gene in *P. aeruginosa* (Rodríguez-Rojas et al., 2009; Le et al., 2014). Some of the roles described for pyomelanin include bacterial surface attachment, extracellular electron transfer, iron reduction/acquisition, induction of virulence factor expression, heavy metal binding and protection from environmental stress (Hunter and Newman, 2010; Hosseinidoust et al., 2013b). Thus, the arising of brown-pigmented colonies in this context is probably another consequence of the stress caused by phage treatment.

The fact that phage-resistant variants were observed earlier in biofilms than in planktonic cultures (**Figure 2**) remains to be explained. Resistance to antibiotics in a biofilm phenotype can be partially explained by the presence of hypermutable strains (Driffield et al., 2008; Oliver and Mena, 2010). The main cause of hypermutation in *P. aeruginosa* strains is the inactivation of the mismatch repair system, which mostly affects the antimutator genes *mutS*, *mutL*, and *uvrD* (Oliver et al., 2002; Rodriguez-Rojas et al., 2011). We hypothesized that the faster emergence of resistant variants in biofilms compared to planktonic cultures could benefit from the presence of hypermutable *P. aeruginosa* strains. Nonetheless, the genome analysis of the five *P. aeruginosa* variants did not reveal any mutation in those genes. Thus, the mutations observed in biofilms are probably a consequence of the endogenous oxidative stress, which results from the exposure of cells to reactive oxygen species (e.g., hydrogen peroxide and superoxide) generated as by-products of aerobic metabolism (Poole, 2012; Moyano et al., 2014). The oxidative stress leads to DNA damage within biofilms resulting in the development of genetic variants with high adaptability to external conditions (Boles and Singh, 2008), which might explain the results obtained.

Overall, strategies that could limit the rise of phage-resistant bacterial strains should be addressed, such as the combination of phages with other antimicrobial agents. Some works already demonstrated synergistic effects between phages and antibiotics (Ryan et al., 2012; Kamal and Dennis, 2015; Oechslin et al., 2016). Furthermore, in the studies reported by Oechslin et al. (2016), the emergence of phage-resistant mutants *in vitro* was prevented by combination treatment with ciprofloxacin and phage; although phage-resistant mutants were observed in *in vitro* studies, the same did not occur *in vivo*.

## Concluding Remarks

The high genomic plasticity of bacteria confers great ability of rapid adaptation to changing environmental conditions. Thus, it is possible to find a great genetic and phenotypic diversity within a bacterial population and, when a selective pressure is applied, bacterial variants that are better adapted to the environmental changes may outcompete other variants.

In this work, although the emergence of phage-resistant bacterial cells was observed in phage infection experiments both in biofilm and planktonic cultures, in most cases, the arising of *P. aeruginosa*-resistant variants occurred faster in biofilms, possibly due to the increased genetic variability of biofilm cells. Additionally, the use of a phage cocktail resulted in a lower percentage of phage-resistant variants than the individual use of each phage because different phages are able to target different bacterial receptors.

It was further observed in this work that all phage-resistant *P. aeruginosa* variants isolated from biofilm infection with the phage cocktail exhibited reduced motilities compared to wild-type strain. The genomic analysis of five variant strains, which were resistant to the three phages used in this study, revealed mutations or deletions in genes that are essential for the synthesis of both receptor types associated with phages: the *pil* genes and *galU*, which are involved in the synthesis of the type IV pilus and the LPS core, respectively. Moreover, these phage-resistant variant strains are as well fully adapted to the biofilm phenotype as the wild-type strain and the selective pressure of phages toward biofilms did not jeopardize their biofilm formation ability.

## Author Contributions

DP, SS, and JA designed the study. DP performed the experiments. AD performed the genomic analysis of bacteria. YH and JL performed the LPS analysis of bacteria. EA and CK performed the microscopy observations and complementation experiments. DP and JA wrote the manuscript. All authors analyzed the data and reviewed the manuscript.

## Conflict of Interest Statement

The authors declare that the research was conducted in the absence of any commercial or financial relationships that could be construed as a potential conflict of interest. The reviewer EF and handling Editor declared their shared affiliation, and the handling Editor states that the process nevertheless met the standards of a fair and objective review.

## Footnotes

1 http://www.ebi.ac.uk/ena

## Supplementary Material

FIGURE S1 Phage susceptibility assays. **(A)** One drop of phage suspension is placed on the Petri dish and allowed to run down the plate (vertical line). **(B)** Once dry, several bacterial colonies are picked and streaked against the phage (horizontal line). **(C)** After overnight incubation, the susceptibility of the bacterial strains to the phage is evaluated. **(D)** Example of a phage susceptibility assay using phage LUZ19.

FIGURE S2 Number of viable cells present in 24-h biofilms of *P. aeruginosa* PAO1 wild-type (WT) and phage-resistant variant strains (V3, V4, V6, V7, and V10).

FIGURE S3 SDS–PAGE of LPS-banding profiles of *P. aeruginosa* PAO1 wild-type and phage-resistant variant strains (3, 4, 6, 7, and 10) complemented with *galU* gene.
