# Supplementary figures and images for: A Genotypic Analysis of Five P. aeruginosa Strains after Biofilm Infection by Phages Targeting Different Cell Surface Receptors
Source: Front Microbiol. 2017 Jun 30;8:1229. doi: 10.3389/fmicb.2017.01229 (PMC5492357; doi:10.3389/fmicb.2017.01229)

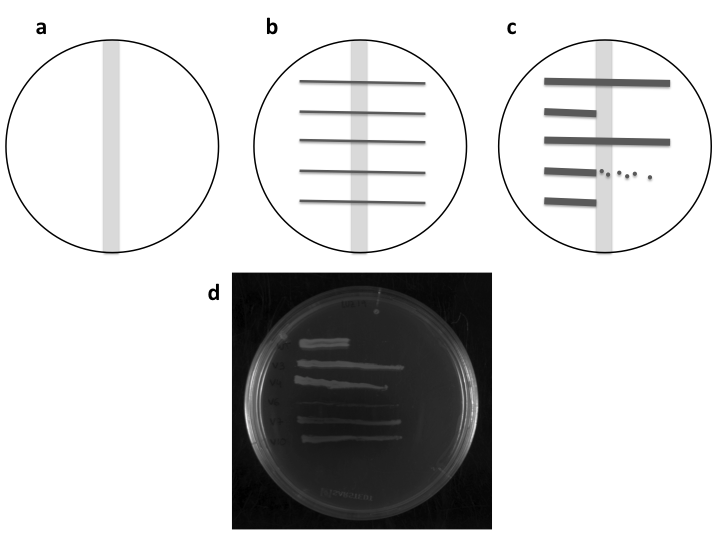

Supplement: FIGURE S1 — Phage susceptibility assays. (A) One drop of phage suspension is placed on the Petri dish and allowed to run down the plate (vertical line). (B) Once dry, several bacterial colonies are picked and streaked against the phage (horizontal line). (C) After overnight incubation, the susceptibility of the bacterial strains to the phage is evaluated. (D) Example of a phage susceptibility assay using phage LUZ19. [file Image_1.TIF]

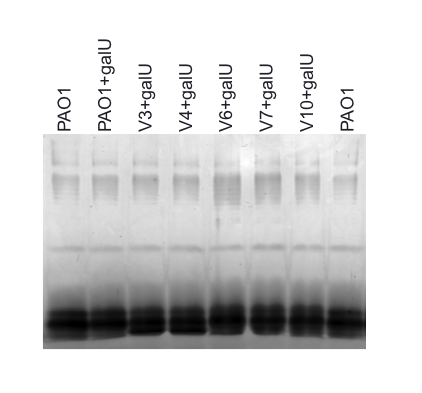

Supplement: FIGURE S3 — SDS–PAGE of LPS-banding profiles of P. aeruginosa PAO1 wild-type and phage-resistant variant strains (3, 4, 6, 7, and 10) complemented with galU gene. [file Image_3.TIF]
